# Mixed methods prospective findings of the initial effects of the U.S. COVID-19 pandemic on individuals in recovery from substance use disorder

**DOI:** 10.1371/journal.pone.0270582

**Published:** 2022-07-01

**Authors:** Katherine Shircliff, Melissa Liu, Christiana Prestigiacomo, Melissa Fry, Kevin Ladd, Misty Kannapel Gilbert, Mary Jo Rattermann, Melissa A. Cyders

**Affiliations:** 1 Department of Psychology, Indiana University Purdue University Indianapolis, Indianapolis, Indiana, United States of America; 2 Department of Sociology, Indiana University Southeast, Indianapolis, Indiana, United States of America; 3 Department of Psychology, Indiana University South Bend, Indianapolis, Indiana, United States of America; 4 LifeSpring Healthcare System, Indianapolis, Indiana, United States of America; 5 Research & Evaluation Resources LLC, Indianapolis, Indiana, United States of America; 6 Community Fairbanks Recovery Center, Indianapolis, Indiana, United States of America; Universita degli Studi di Milano-Bicocca, ITALY

## Abstract

The beginning of the U.S. COVID-19 pandemic interrupted integral services and supports for those in recovery from substance use disorders. The current study used qualitative and quantitative data to identify 1) pandemic-related barriers/stressors, 2) coping strategies employed, and 3) how the stressors and strategies predicted subsequent substance use frequency. Participants were 48 adults (40.5% female; 90.2% White) between 26 and 60 years old (M = 42.66, SD = 8.44) who were part of a larger, multi-year longitudinal study of individuals in recovery from substance use disorders. Individuals completed two interviews, one during the six weeks of initial stay-at-home orders in the state in which data were collected and the second within six to twelve months of their initial interview. Common barriers to recovery included cancelled support meetings, changes in job format (i.e., being fired or furloughed), and lack of social support. Common coping strategies included self-care, leisure activities/hobbies, taking caution against exposure, and strengthening personal relationships. The relationship between cravings at baseline and substance use at follow up was stronger for those who experienced worsening of their mental health (B = 21.80, *p* < .01) than for those who did not (B = 5.45, p = 0.09), and for those who were taking caution against exposure (B = 24.57, p < .01) than for those who were not (B = 1.87, *p* = 0.53). Those who engaged in self-care (B = 0.00, p>.99) had lower rates of substance use at follow-up than those who did not employ self-care as a coping mechanism (B = 16.10, *p* < .01). These findings inform research priorities regarding prospective effects of the pandemic on treatment endeavors, particularly emphasizing treating mental health and encouraging self-care strategies.

## 1.0 Introduction

Successful recovery from a Substance Use Disorder (SUD) is facilitated by access to treatment professionals and regular healthcare, social-support, routine, and self-purpose [1–4]. The COVID-19 pandemic in the U.S. disrupted these conditions, when state-mandated closings started in mid-March 2020 and extended up to 6–8 weeks in some states [5]. These changes left those with SUD potentially vulnerable to adverse consequences [6]. Hospital systems initially restricted care to only those deemed “essential,” limiting access to support groups and behavioral treatments; although, many in-person care settings shifted to virtual environments after a period of adjustment [7]. Access to opioid agonist treatment for Opioid Use Disorder likewise was reduced at the beginning of the pandemic due to limited resources to continue required monitoring [8, 9]. The Substance Abuse and Mental Health Services Administration [10] subsequently issued guidelines for increased flexibility in existing opioid agonist monitoring policies during the COVID-19 pandemic, reducing COVID-related barriers to such treatments. Individual states made additional changes to SUD care during the pandemic, particularly by increased access to telehealth and online supports [11]. Despite these modifications, early interruptions in access may have precipitated long-reaching effects on SUD recovery and relapse.

Cross-sectional research documented how early restrictions (e.g., decreased access to social supports and limited time engaged in drug-free hobbies) during the COVID-19 pandemic were related to greater drug craving [12]. Craving, the subjective experience of wanting to use a drug [13], is widely experienced by those with SUD and serves as a criterion for a substance use disorder diagnosis [14]. Craving is also a major predictor of relapse [15]; in samples of alcohol- [16, 17] and opiate-dependent [18] adults seeking treatment, cravings significantly predicted substance use at follow-up. The relationship between substance cravings and later use can be reduced via a multitude of moderators, such as mindfulness and avoidance coping [19, 20].

Before the onset of the COVID-19 pandemic, the United States was already in the midst of an existing substance use crisis. Between 2013 and 2019, death from synthetic opioid use increased 1,040% [21]. This exponential increase in death from substance use was exacerbated by the onset of the COVID-19 pandemic, as deaths from substance use increased by 29.4% in 2020 alone [22]. Within the first eight months of onset of the pandemic, treatment providers reported an increase in patients with SUD seeking care, and only half of clinic appointments offered telemedicine services [23]. In early April 2020, one study [24] found that use of alcohol, cannabis, sedatives, and prescription opioids had increased and use of illicit opiates, amphetamine, and cocaine had decreased across the globe. Additionally, perceived supply of substances decreased, while the price of cannabis, opiates, amphetamines, and cocaine increased. Approximately 13% of adults reported new or increased substance use due to stress from the pandemic [25], likely driven by attempts to cope by using substances [26]. Contextualizing variables, such as worry about the pandemic [27], perception of susceptibility to COVID-19 infection [28], and experiences with isolation [29], may also increase stress for those in recovery from SUD, thus increasing attempts to cope via substances.

Limited research has examined the prospective effects of these early barriers on later recovery management during the pandemic. One mixed methods study [30] found that women in recovery experienced multiple barriers, such as limited access to social relationships, housing, employment, and health care, but maintained their sobriety over the first few months of the pandemic. Multiple qualitative reviews have highlighted the importance of these disruptions to care for people in SUD recovery [31–33]; but little empirical, prospective research has been conducted to date.

The goal of the current study is to prospectively document how early pandemic-related barriers and facilitators of recovery influenced later substance use among a sample of individuals in SUD recovery. A more thorough understanding of early barriers in the initial weeks of the COVID-19 pandemic, during the immediate and most drastic changes of the state-mandated closings, allows for the identification of factors that may be important markers for ongoing recovery throughout the COVID-19 pandemic. Data collection occurred in two waves: during the first six weeks of the stay-at-home orders in the state in which the data were collected, when the largest disruptions to care occurred [34], and again within a six-to-twelve month follow-up period (see a timeline of COVID-19 events and data collection in S1 Fig). The period immediately following state mandated stay-at-home orders was a time in which substance use increased significantly [35] and individuals in recovery were at their most vulnerable to remission of use [36]. The second time period corresponded roughly with the peak number of daily deaths from COVID-19 in the Midwest state in which the data were collected [37]. Though this timeline allowed for greater variability in participant experiences at follow-up (e.g., introduction of vaccines, mask mandates, etc.), the two waves of data collection allowed for a snapshot into two times in which the pandemic was potentially at its most disruptive states.

The current study utilizes both quantitative and qualitative data in a sample of individuals who received treatment for SUD. The majority of work to date has focused solely on barriers to recovery amid pandemic-related closures; the current study aimed to expound upon previous work by additionally focusing on ways those in recovery effectively coped with this stressful experience, as these could inform strategies for future recovery planning. The specific goals of this study were to use qualitative and quantitative data to identify 1) barriers/stressors brought on by the pandemic, 2) coping strategies employed, and 3) how the stressors and strategies predicted subsequent substance use frequency. We hypothesized that 1) individuals in recovery would report increased barriers to recovery during the beginning of the COVID-19 pandemic and that 2) reported barriers and coping strategies would moderate the effect of early substance cravings on later substance use frequency at follow-up, such that barriers would strengthen the relationship between baseline cravings and later substance use frequency, whereas coping would weaken the relationship.

## 2.0 Method

### 2.1 Participants

Participants were part of a larger, mixed methods longitudinal study with the goal of identifying facilitators and barriers to long-term recovery from substance use disorders. The parent study is a multi-site three-year longitudinal study of 350 people in recovery recruited from local addiction treatment centers. Funded by a large university grant, the goal of the parent study is to characterize recovery-related experiences, including treatment accessibility and utilization, barriers to treatment, and differential trajectories of treatment course. Individuals were eligible to participate if they reported being in recovery from any Alcohol or Substance Use Disorder and participants completed semi-structured interview and questionnaire reports every 3–6 months while enrolled in the study. Inclusion criteria for the parent study were ≥18 years old, able to understand English, self-identify as in “recovery” (i.e., report an intent to actively manage the symptoms of their substance use disorder), and receiving treatment at one of the two participating addiction treatment centers. The current study included participants who completed an interview within six weeks from the start of state-specific stay-at-home orders (roughly from the end of March-early May 2020) and one follow-up interview 6–12 months later (roughly from July 2020 to June 2021).

### 2.2 Measures

#### 2.2.1 Sample characteristics

Sample characteristics assessed included gender, age, race, ethnicity, substances used and treatment history.

#### 2.2.2 The COVID-19 interview instrument

Included questions concerning the initial effects of the COVID-19 pandemic on everyday life, recovery/treatment plans, and substance use/cravings. The questionnaire was created by the research team and included self-reported qualitative (e.g., “How has the COVID-19 pandemic affected your everyday life,” “How has the COVID-19 pandemic affected your recovery or treatment plan,” and “Have you noticed any changes in substance use cravings?”; total of 10 questions) and quantitative (e.g., using scalar ratings; “How worried are you about the COVID-19 pandemic?”; total of 3 questions) items. In total, the COVID-19 instrument required about 5–10 minutes to complete. COVID-19 interview questions are included as supplemental material (S1 Table).

#### 2.2.3 Substance use cravings

Were based on DSM-5 criteria and measured on a Likert scale (not at all (1), a little bit (2), somewhat (3), quite a bit (4), very much (5)) in response to two craving-related statements–“In the past three months, my desire to use drugs was overpowering” and “In the past three months, I craved drugs.” These two items were averaged to create an overall score, with higher scores indicating higher substance use cravings (coefficient alpha = 0.91 in the current sample).

#### 2.2.4 The timeline follow-back

(TLFB; [38]) measured substance use over the previous 90-days. Guided by a trained research assistant, participants reported the days over the previous 90 in which they drank alcohol and the number of standard alcoholic beverages consumed on each occasion. In addition, participants reported the number of days in the previous 90 when they used any other illicit substance. Substance use frequency was calculated as total number of days in which any substances and/or alcohol were used (not including nicotine; “days using substances”).

### 2.3 Procedures

Participants were recruited from two addiction treatment centers in a midwestern state: one located in an urban region that primarily serves patients with private insurance and the other located in a rural region that provides service to patients mainly covered by Medicaid. Both facilities provide detox, inpatient, and outpatient services. Due to the remote nature of the interviews, the institutional review board granted a waiver of documentation of informed consent. Each participant was read and e-mailed a copy of the informed consent document and was given an opportunity to ask questions about participation. Participants then gave verbal approval of participation, which was noted by a member of the research team on each participant’s informed consent document. Phone interviews were conducted at both initial interview and follow-up (6–12 months following initial interview), and participants received $40 in the form of a check or gift card for the initial interview and $20 for follow-up interview completion. The study was approved by the local institutional review board at the sponsoring university (IRB #1810016238).

#### 2.3.1 Data analysis

Qualitative responses were recorded by two independent coders and themes were identified by the first author of this report, who was trained in thematic analysis. Quantitative statistical analyses were conducted using SPSS ver.27. In order to assess how individuals managed their recovery during the initial weeks of the pandemic, we coded qualitative answers from the COVID-19 Interview Instrument for general attitudes regarding the pandemic and performed descriptive analyses of the quantitative items, along with independent samples t-tests across timepoints. We also conducted a series of paired samples t-tests to assess changes in substance use variables across baseline and follow-up. To examine whether the identified barriers and coping strategies moderated the relationship between baseline substance cravings and follow-up substance use frequency, we conducted a series of moderation analyses using the PROCESS macro [39] with baseline substance cravings as the independent variable, follow-up days using substances as the dependent variable, and each barrier or coping strategy as the moderator. Significant interactions, at a threshold of *p*<0.05, were probed and graphed to examine patterns of relationships.

## 3.0 Results

### 3.1 Sample characteristics

The final sample included 48 individuals (40.5% female; 90.2% White; 77.1% Heterosexual) between the ages of 26 and 60 (M = 42.66, SD = 8.44); most participants were under 1 year in treatment (68.8%) and used depressants (e.g., alcohol; 80.9%) (see Table 1 for full sample characteristics).

**Table 1:** Sample characteristics (N = 48).

Demographics	N (%)
Age (M (SD))	42.66 (8.44)
Female	17 (40.5%)
White	37 (90.2%)
Non-Hispanic or Latinx	36 (75.0%)
Straight/Heterosexual	37 (77.1%)
**Previous Treatment History**	
Inpatient	33 (67.3%)
Outpatient	22 (44.9%
Detox	4 (8.2%)
Sober Living Program	2 (4.1%)
Other	4 (8.2%)
**Substance Endorsements**	
Stimulant	21 (44.7%)
Depressant	38 (80.9%)
Opioid	19 (40.4%)
Cannabis	25 (53.2%)
**Total Length in Treatment**	
Under 1 year	33 (68.8%)
1–3 years	4 (8.3%)
3–5 years	1 (2.1%)
5+ years	1 (2.1%)
Not enough information	9 (18.7%)
**Medication Assisted Treatment (MAT)**	
Currently on MAT	15 (31.9%)
Methadone	0 (0.0%)
Suboxone	10 (66.7%)
Vivitrol	3 (20.0%)
Naltrexone	2 (13.3%)

### 3.2 Pandemic experiences

#### 3.2.1. Pandemic experiences at baseline

Individuals reported worry about the COVID-19 pandemic at an average of 54.68/100 (SD = 29.84) and self-reported their perceived likelihood of being infected with COVID-19 as 58.00/100 (SD = 31.17) (Table 2, left column). Sixty-five percent rated their stress during the pandemic as “worse” or “much worse.” Worries about COVID-19 susceptibility and transmission were reported across the sample: 66.7% reported that they, or someone they are close to, had a pre-existing condition that made them more susceptible to the virus, 15.0% reported previous COVID-19 infection; 70.8% reported being quarantined or isolated, with 14.3% of that group in quarantine alone.

**Table 2:** Management of recovery at initial interview and follow-up.

	Initial Interview	Follow-up	Comparison
Worry about the pandemic, M(SD)	54.68 (29.84)	53.00 (29.33)	*t* = -0.28, *p* = 0.78
Likelihood of infection, M(SD)	58.00 (31.17)	56.37 (30.14)	*t* = 0.08, *p* = 0.93
Changes in cravings, N(%)	13 (27.1%)	9 (29.0%)	*X*^*2*^ (4, 30) = 4.85, *p* = 0.30
Increases, N(%)	8(16.7%)	3(9.7%)	
Decreases, N(%)	5 (10.4%)	6(19.4%)	
No change, N(%)	35 (72.9%)	22(71.0%)	
Cravings scale scores, M(SD)	2.36(1.44)	1.48(0.52)	*t* = 2.53, *p* = 0.01
My desire to use drugs seemed overpowering	2.40 (1.52)	1.46(0.76)	*t* = 2.49, *p* = 0.02
I craved drugs	2.33(1.49)	1.50(0.51)	*t* = 2.39, *p* = 0.03
Currently using substances, N(%)	13(27.1%)	4 (10.8%)	*X*^2^ (2, 36) = -20.13, *p*<0.01
Increases, N(%)	2 (15.4%)	3(8.1%)	
Decreases, N(%)	0 (0.0%)	0(0.0%)	
No change, N(%)	11(84.6%)	1(2.7%)	
Days using substances, M(SD)	11.35 (18.56)	9.57(26.25)	*t* = 0.29, *p* = 0.39

Note: Worry about the pandemic and likelihood of infection are scaled 0%–100%, with higher scores indicating more worry and increased perception of infection likelihood. Substance cravings are measured on a Likert scale (from not at all(1) to very much(5)), with higher scores indicating more cravings for substances. Days using substances are out of the previous 90 days.

#### 3.2.2 pandemic experiences at follow-up

Follow-up participants reported worry about the COVID-19 pandemic at an average of 53.00/100 (SD = 29.33) and rated their likelihood of COVID-19 infection as 56.37/100 (SD = 30.14). A dependent samples t-test confirmed no significant differences between worry (*t* = -0.28, *p* = 0.78) or perceived likelihood of infection (*t* = 0.08, *p* = 0.93) between baseline and follow-up (Table 2, left column). Worry about COVID-19 susceptibility and transmission were reported across the sample: 66.7% reported that they, or someone they are close to, had a pre-existing condition that made them more susceptible to the virus, 9.1% of individuals were infected with COVID-19 since initial interview; 24.2% reported being quarantined or isolated, with 25.0% of that group in quarantine alone.

### 3.3 General recovery management

#### 3.3.1. Recovery management at baseline

Substance cravings were reported as a mean of 2.36/5; 16.7% reported higher substance use cravings and 10.4% reported fewer cravings since the start of the pandemic (Table 2, left column). On average, participants reported using any substances on 11 in the previous 90 days (M = 11.35, SD = 18.56, range 0–61). Twenty-seven percent of the sample reported current use of substances; of these, 15.4% reported increases and 84.6% reported no changes in use since the start of the pandemic (Table 2, left column). COVID-related worry, substance craving, and substance use did not differ significantly across age, gender, or race (*p’s*>0.10) at baseline. Days using substances differed significantly across age (*p*<0.01), such that older individuals reported fewer days using substances. Initial substance cravings were significantly correlated with days using substances (*r* = 0.78 *p*<0.001) at baseline.

The majority (85.4%) of participants expressed frustration and stress regarding pandemic-related closures. Some participants noted, “Quarantine makes me depressed and very irritable and aggressive with people, like I’m going crazy,”“A lot has shut down and meetings have been cancelled. I’ve been scrambling to make accommodations but they keep falling through,” and “I can’t do AA meetings, I can’t go to therapy in person, I can’t join a halfway house. I’m not getting enough support to really get my life back on track.” However, a small group of individuals (6.3%) reported that isolation minimized opportunities to use (e.g., “The end of the constant party in the backyard has stopped my temptations to drink.”).

#### 3.3.2 Recovery management at follow-up

Since the baseline interview, 71.0% of the sample reported no changes in substance use cravings, 19.4% reported fewer cravings, and 9.7% reported increases in cravings (Table 2, center column). Level of substance craving was reported as 1.48/5(SD = 0.52); significantly lower at follow-up than at baseline (*t* = 2.53, *p* = 0.01). Four individuals (10.8% of the sample) reported that they were using substances at their follow-up interview; of these, 3 individuals (8.1%) reported increases in use and 1 individual reported no change (2.7%) (Table 2, center column). A chi square analysis revealed that substance use prevalence was significantly lower at follow-up as compared to baseline (*X*^2^ (2, 36) = -20.13, *p*<0.01). On average, individuals reported using substances on 9.57 days (SD = 26.25, range 0–90) and this value did not significantly differ from baseline use frequency (*t* = 0.29, *p* = 0.39). At follow-up, COVID-related worry differed across age, F(17) = 3.60, p<0.01. Substance cravings and substance use did not differ significantly across age, gender, or race (*p’s*>0.20) at follow-up. The relationship between substance craving and substance use frequency at follow-up (*r* = 0.35 *p* = 0.08) fell just short of significance.

During follow-up, 61.3% of participants reported continued attendance at virtual meetings and 29.0% reported no longer attending meetings or decreased meeting attendance (e.g., “I haven’t continued meetings or any other recovery-oriented activities outside of my medication assisted treatment.”). Twenty-two percent of participants reported attending virtual meetings that were in the process of shifting back to being in person (e.g., “I had initially done zoom meetings, but we are starting to meet in person.”). A small group of participants (12.9%) reported no change in their treatment since the initial interview. Two participants noted that they had been doing better in their recovery since the initial interview, with one who stated, “I have been going to more meetings, 2 to 3 meetings each day at most,” and another stated, “This actually works better for me, so I don’t have to drive on my days off.”

### 3.4 Pandemic-related barriers

#### 3.4.1 Reported pandemic-related barriers

Participants described a number of barriers, including interruptions in practices that support recovery. One individual stated, “My recovery is dependent on structure—the pandemic uprooted every routine I had going.” Changes in structured routines such as employment (e.g., “I couldn’t work, so I just kept using drugs.”), and treatment (e.g., “My halfway house is being shut down and I have to move out soon.”) were reported. Though participants described reduced access to therapy and support groups, individuals on medications for Opioid Use Disorder note continued access to treatment, stating “I’ve still been able to get vivitrol” and “my suboxone has continued,” though, one individual noted a delay in access, stating, “I was late on my vivitrol shot due to being quarantined.”

Upon consolidation, seven themes emerged concerning barriers to recovery due to the onset of the pandemic (Table 3): 1) 12-step support meetings cancelled, shifted online, or changed in any facet (70.8%); 2) being fired, furloughed, or experienced any change in job format (e.g. work from home and shift in job responsibilities) (47.9%); 3) lessened social support from family, friends, and overall decreased sense of community (41.6%); 4) decreased opportunity for leisure activities (e.g. exercise, social events, and hobbies) (37.5%); 5) decreased access to treatment outside of 12-step meetings (e.g., closed recovery houses, interrupted therapy, availability of treatment programs etc.) or inability execute treatment plans (31.2%); 6) changes in personal schedule (e.g., new full-time care of a child or newfound free time; 31.2%), and 7) worsened mental health (e.g., feelings of being overwhelmed, anxious, or depressed; 29.1%).

**Table 3:** Themes of barriers to recovery with representative quotations.

Barrier Theme (% endorsed)	Representative Quotation
Meetings cancelled, shifted to online, or changed (70.8%)	*“A lot has shut down [and] meetings [have been] cancelled*.*”*
	*“[I’m] scrambling to make accommodations but it keeps falling through*. *I haven’t engaged because it seems so noncommittal*.*”*
Change in job format (47.9%)	*“[I’ve been furloughed from] my job of 9 years in food service at a hotel*, *with no return date in sight”*
	*“I’ve been furloughed with no pay”*
	*“I’ve lost my job and my insurance”*
Lack of socialization or social support (41.6%)	*“I’ve been isolating more”*
	*“Trying to maintain a community feel [in meetings] is very important”*
	*“[I] can’t stay in contact with a sponsor or communicate with the recovery community”*
Lack of opportunity for leisure activities (37.5%)	*“I can’t get out to do anything*. *I can’t even get a date anymore”*
	*“I can’t even go out to get a haircut”*
	*“I can’t shoot pool or exercise”*
	*“Everything is closed*, *so I can’t go out or enjoy leisure activities”*
Inability to put treatment plan into place (31.2%)	*“It’s been difficult trying to find a sponsor”*
	*“[They’ve] canceled level 1 meetings [and there are] no more group sessions”*
	*“[I’ve been] dismissed from [treatment facility’s] classes*, *regardless of completion”*
	*“This put every plan I made in rehab on hold”*
	*“This has prevented access to programs I need for recovery”*
Change in schedule that supports recovery (31.2%)	*“I’m just bothered about not knowing what do to”*
	*“I have no money and a ton of free time”*
	*“I couldn’t work so I started using drugs”*
	*“I’ve been trying to stay busy and avoid idle time”*
Mental health (29.1%)	*“[I’ve been feeling] very anxious”*
	*“Quarantine makes me depressed and very irritable and aggressive with people and [I feel] like I’m going “crazy*.*”*
	*I couldn’t get unemployment and am stressed out”*
	*“[I’m]really falling into a depression spiral”*

#### 3.4.2 Interactions between barriers and cravings

**Fig 1:**
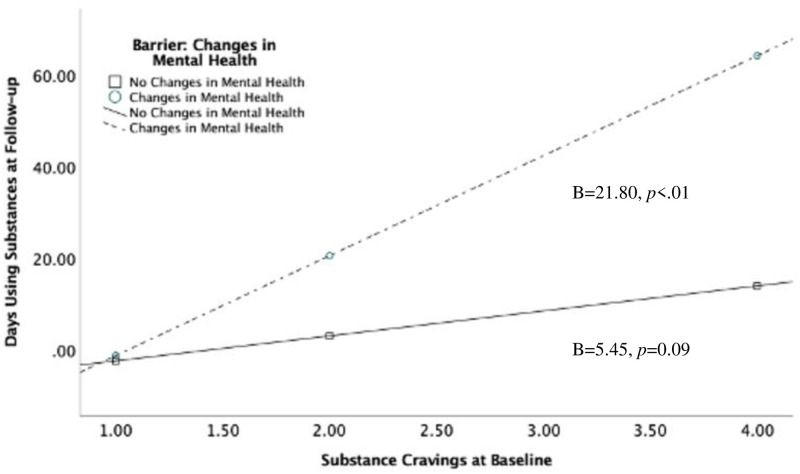
Interactions between baseline substance cravings and changes in mental health to predict later days using substances (n = 48).

There was a significant interaction between baseline substance cravings and worsened mental health (B = 16.34, *p* = 0.02), such that the relationship between cravings and days using substances at follow-up was stronger among those who experienced worsened mental health (B = 21.80, *p* < .01) than those who did not (B = 5.45, p = 0.09) (Table 4; Fig 1, right). There were no other significant interactions with the remaining barriers (support meetings changed, change in job format, decreased social support, decreased leisure activities, decreased access to treatment, and changes in personal schedule).

**Table 4:** Interaction between baseline substance cravings and changes in mental health to predict days using substances at follow-up.

	*B*	*SE*	*t*	*p*-level
Constant	-7.84	8.39	-0.93	0.35
Substance Cravings at Baseline	5.45	3.14	1.73	0.09
Barrier: Changes in Mental Health (0,1)	-15.19	16.37	0.92	0.36
Craving X Barrier Interaction	16.34	6.82	2.39	0.02
Endorsed Changes in Mental Health	21.80	6.05	3.59	<0.01
Did not endorse	5.45	3.14	1.73	0.09

### 3.5 Pandemic-related coping

#### 3.5.1 Reported pandemic-related coping

Qualitative responses showed that, for many, isolation was an opportunity to reconnect with friends and family members (e.g., via phone and video calls) and engage in self-care hobbies (e.g., journaling, praying, cooking, and spending time outside). One individual highlighted the ability to attend more meetings via online formats. Another participant noted the importance of previous treatment in supporting resiliency: “[My treatment facility] and Alcoholics Anonymous have prepared me very well for this time.”

Upon consolidation, four themes of coping emerged (Table 5): 1) self-care (including exercise, positive talk, reflection and prayer) (50.0%), 2) leisure activities and hobbies (39.5%), 3) caution and following CDC guidelines (i.e. wearing a mask and social distancing) (33.3%), and 4) strengthening personal relationships (25.0%).

**Table 5:** Themes of coping strategies that facilitate recovery with representative quotations.

Coping Mechanism Theme (% endorsed)	Representative Quotation
Self-care (e.g., exercise, reflection/prayer, positive talk; 50.0%)	*“Trying to make the best of the situation and stay positive*.*”*
	*“Praying about things and trying to stay positive because things have been better with my recovery*.*”*
Leisure activities and hobbies (39.5%)	*“{I’m] finishing projects I hadn’t been able to do before”*
	*“I’ve been working on project*, *cleaning around the house*, *[being] active with my wife*, *housework*, *and writing creatively”*
Being cautious/ following CDC guidelines (33.3%)	*“I’ve been extra cautious about being around people*, *sanitizing everything*, *and using grocery delivery”*
	*“[I’m] following directions*, *staying inside*, *wearing my mask*, *and trying to keep my immune system up*.*”*
	*“[I’m] wiping off door handles and washing my hands”*
Strengthening personal relationships (25.0%)	*“[I have] connected with old friends that I wouldn’t normally have time to do”*
	*“[I am] continuing to keep up personal relationships through phone or zoom meetings”*
	*“I have been engaging in new hobbies with my son*.*”*

#### 3.5.2 Interactions between coping and cravings

**Fig 2:**
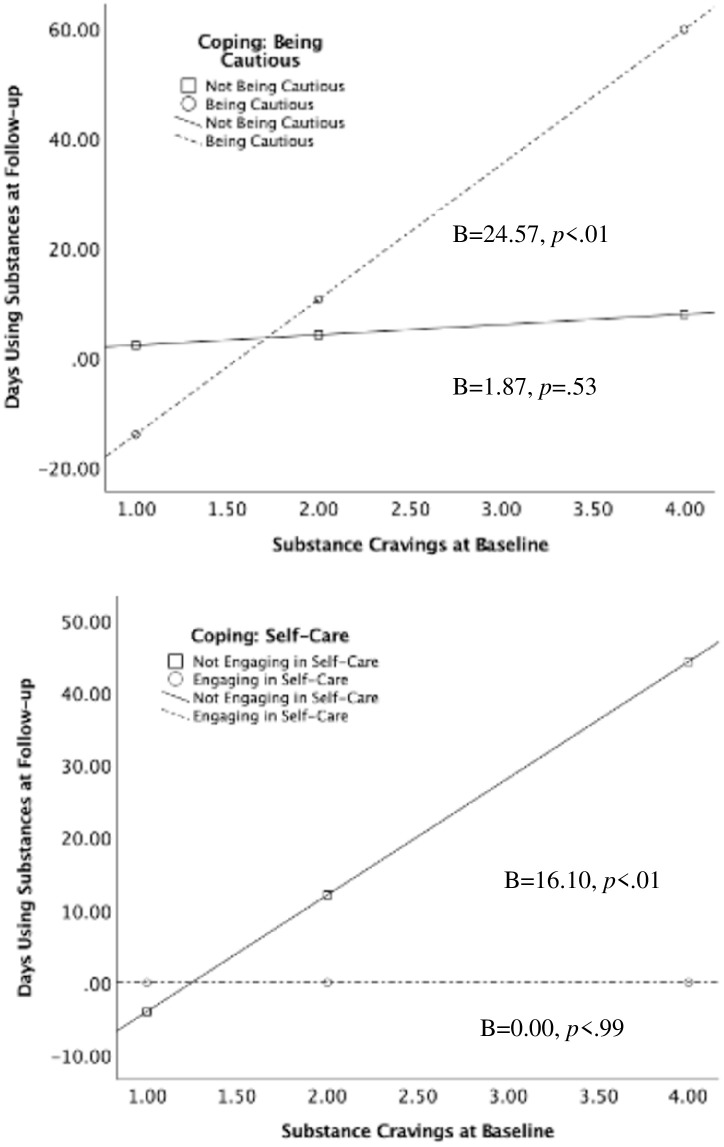
Interactions between baseline substance cravings and coping strategies to predict later days using substances (n = 48). Top panel depicts the interaction with being cautious and Bottom panel depicts the interaction with self-care.

There was a significant interaction between baseline substance cravings and being cautious/following CDC guidelines (B = 22.69, *p*<0.01), such that the relationship between cravings and days using substances at follow-up was stronger among those who were taking caution against infection (B = 24.57, p < .01) than those who were not (B = 1.87, *p* = 0.53) (Table 6; Fig 2, left). There was also a significant interaction between baseline substance cravings and self-care (B = -16.10, *p*<0.01), such that the relationship between cravings and days using substances at follow-up was stronger among those who did not engage in self-care (B = 16.10, *p* < .01) than those who did (B = 0.00, p>.99) (Table 7; Fig 1, right). There were no other significant interactions with the remaining coping strategies (engaging in hobbies and strengthening personal relationships).

**Table 6:** Interaction between baseline substance cravings and being cautious to predict days using substances at follow-up.

	*B*	*SE*	*t*	*p*-level
Constant	0.39	7.48	0.05	0.95
Substance Cravings at Baseline	1.87	2.95	0.63	0.53
Coping: Being Cautious (0,1)	-38.94	14.92	-2.61	0.01
Craving X Coping Interaction	22.69	5.66	4.00	<0.01
Endorsed Being Cautious	24.57	4.83	5.08	<0.01
Did not endorse	1.87	2.95	0.63	0.53

**Table 7:** Interaction between baseline substance cravings and self-care to predict days using substances at follow-up.

	*B*	*SE*	*t*	*p*-level
Constant	-20.11	10.36	-1.93	0.06
Substance Cravings at Baseline	16.10	3.92	4.09	<0.01
Coping: Self-Care (0,1)	20.11	14.20	1.41	0.16
Craving X Coping Interaction	-16.10	5.52	-2.91	<0.01
Endorsed Self-Care	0.00	3.88	0.00	>0.99
Did not endorse	16.10	3.92	4.09	<0.01

## 4.0 Discussion

This study combined qualitative and quantitative data collected during the first six weeks of COVID-19-related stay-at-home orders, and within 6–12 months following this period, to characterize how individuals in recovery from SUD have coped during the COVID-19 pandemic, as well as to document early barriers and facilitators of recovery that might have influenced later substance use. The combination of qualitative and quantitative approaches allowed themes to emerge from participant interviews, and provided initial quantitative patterns of effects to inform future research. Across both our qualitative and quantitative data, participants reported high worry and frustration during this time. Although isolation was largely viewed as a barrier to treatment, it was also reported to be helpful in avoiding opportunities for substance use for a small minority of individuals. Overall, substance use and craving remained relatively stable or decreased across the two time points in this recovery sample. Common early barriers included treatment interruptions, employment changes, decreased social support, lack of opportunity for leisure activities, schedule interruptions, and changes in mental health. Common effective coping strategies included self-care, leisure activities, being cautious, and strengthening personal relationships. Worsened mental health, taking caution against infection, and engaging in self-care influenced how participants responded to substance cravings.

Overall, a majority of participants reported high worry during the initial weeks of the U.S. COVID-19 pandemic. Elevations in stress, a well-known contributor to substance relapse, can exacerbate substance use cravings [40]. Indeed, initial reports suggest a 17.59% increase in suspected opioid overdose between the weeks leading up to (1/1/2020 through 3/18/2020) and following state-mandated stay-at-home orders (3/19/2020 through 5/19/2020) [5], the time period that overlaps with the period of the first wave of our study. In the current study, substance use frequency rates stayed relatively stable over the follow-up period, while substance cravings and substance use prevalence reduced in this time, suggesting no worsening of this risk over the first year of the pandemic. People in our sample remained largely abstinent (i.e., only 27.1% of our sample reported substance use during these initial weeks and 21.6% at follow-up), at rates lower than those reported prior to the pandemic (40–60%) [41]. These findings are contradictory to previous findings that suggested overall increases in substance use in response to the COVID-19 pandemic [42]. This could be due to the sample used in the current study, as approximately 69% of the sample had been recently discharged from treatment at baseline. Those who have recently completed treatment typically experience weaker cravings and have stronger coping skills than their untreated counterparts [12]. Additionally, as early stages of recovery tend to be the most challenging and hectic [43], our sample may already have been in a state of heightened anxiety and worry about the future when the pandemic began. We hypothesize that our findings suggest that individuals in recovery initially coped well with changes brought on by the pandemic due to the presence of effective coping strategies, many that they learned through their substance use treatment programs.

Individuals reported multiple barriers to treatment during this initial period of stay-at-home orders, most notably the interruption of treatment and social support, which are critical for maintaining recovery [44]. The majority of respondents indicated that they were attending virtual support meetings; however, over a fourth of the sample reported that they no longer attended meetings due to cancelations, perceived reductions in effective “personal connections,” or because available meeting formats felt “chaotic” and “not the same.” Surprisingly, interruptions in meetings did not significantly influence the relationship between craving and subsequent substance use. This likely suggests that many were able to successfully make the transition to online support meetings [45]. Thus, although some in our sample reported online meetings not being as effective as in person meetings, this finding suggests no significant influence on how cravings influenced later substance use. This means that, even if not in the ideal form, social support meetings are likely an important piece of treatment to maintain during unexpected treatment interruptions. This is consistent with other work suggesting effectiveness of online meetings [46, 47], the increased accessibility of such meetings during the pandemic [48, 49], and the need to continue to increase such access. Although access to such meetings has increased, some groups (e.g., 12-step and mutual/self-help groups) may still experience barriers to online meeting engagement, including decreased comfort or privacy to engage or access to reliable internet access, which may increase relapse risk for already underserved populations.

Under a third of the sample reported at initial interview that the pandemic either incited (e.g., “For the first time in 25 years, I have felt a sense of hopelessness”) or exacerbated (e.g., “I have tremendous anxiety, and the pandemic has really heightened it”) negative mental health challenges. The negative impacts of the pandemic on mental health may have persisted even after restrictions were being lifted. These maintained challenges may have lowered participants’ ability to resist urges to use substances, as demonstrated in our qualitative data (e.g., “I have more desire to drink than before all this happened,” and “I used the isolation as an excuse to get high.”). This was also present in our quantitative data, where there was a stronger effect of substance cravings on subsequent use for those with worsened mental health challenges during this time. This combination of increased cravings with persistent worsening of mental health is a perfect storm contributing to substance use in this sample.

Endorsing taking precaution against infection (e.g., physical distancing and isolation) during the early stages of the pandemic strengthened the relationship between substance cravings and later substance use frequency. While being cautious was described by our sample as a coping mechanism, our quantitative data present a more nuanced understanding of this strategy, including potential negative effects. Although isolation may have served as a coping mechanism in that it kept individuals physically safe, doing so may have also had inadvertent negative psychological effects. Those who reported being cautious against infection in the initial interview coupled it with increased emotional distance from integral supports and worsened ability to do normal daily activities (e.g., “I’m being extra cautious around people, sanitizing everything, and doing grocery delivery.”). Additionally, these interruptions may have offset previously established mechanisms for coping with substance use cravings (e.g., “I can’t go to in person meetings, and that’s been a huge setback.”). Routines are an important factor in recovery maintenance [3] and while shifts in some routines produced positive effects [50] such as interruption to routines that previously encouraged their substance use (e.g., “In a way it helps, you can’t go wherever you want”), the majority of our sample reported that the pandemic interrupted routines that supported recovery maintenance (e.g., “My recovery supports have almost all been cancelled”).

Among the many coping strategies endorsed to effectively manage pandemic related changes, self-care practices were particularly important in our study: Self-care practice was the only coping strategy to reduce the effect of substance cravings on later substance use frequency. The qualitative data highlighted a number of different forms of self-care engaged in by our participants, including creative writing or journaling, housework, exercise, and thinking positively. This is consistent with previous work showing clinical utility of self-care strategies in reducing subsequent substance use through increasing engagement in substance-free habits [51–53]. Interestingly, no one who reported engaging in self-care was using substances at follow-up, regardless of their level of substance craving at baseline. The current research supplements existing support for self-care habits and suggests that positive effects of self-care practices during the early stages of the pandemic were sustained over time, as supported by previous work [54, 55], stressing the importance of emphasizing and encouraging such forms of coping for those with SUD to guard against the influence of substance cravings.

Interestingly, participants rated their likelihood of COVID-19 infection as almost twice that of U.S. adults in general (58.0% in the current sample vs. 30.0% in a general adult sample) [56]. This heightened perception of susceptibility may reflect reality, as data suggest that the pandemic is disproportionately affecting the physical and mental wellbeing of individuals who use substances [57, 58] Individuals who use substances may be more susceptible to infection and COVID-related complications due to the negative effects of substance use on immune, respiratory, and pulmonary systems [59–61] and to increased odds of hospitalization, ventilator use, mortality [62], and drug-drug interactions. Additionally, withdrawal from opioids or opioid agonist medications can mimic or mask COVID-19 symptoms [63]. In response to these increased challenges, an international group of experts in addiction medicine and infectious disease published recommendations for care of individuals who have comorbid SUD and COVID-19 diagnoses. These recommendations highlight the importance of early identification of COVID-19 among those in isolation, interrupting COVID-19 transmission where treatment requires in-person care, providing appropriate care for individuals at different stages in their recovery, addressing comorbid medical issues, and minimizing negative social factors, such as homelessness [64], that may increase risk for infection [65, 66]. Despite rating risk of infection as high, only one-third of our sample coped by taking precaution against infection; this means that the majority of our participants did not, which is a worrisome number given the overall increased risk.

### 4.1 Future directions

We see these findings as preliminary, but important, for future research planning. Given the limited research documenting prospective effects of early pandemic-related factors on later substance use, these findings contribute to a nascent, but growing field. Of note, some of our prospective findings do not corroborate previously reported cross-sectional findings, further emphasizing the importance of continued longitudinal study. Given the rapidly changing nature of the pandemic, it is unsurprising that patterns and relationships may have evolved as the pandemic progressed. The timeline of the current project corresponds with two timepoints at which the pandemic may have been at its most disruptive state (initial closures and peak death tolls), which may have contributed to variability in participant experiences and contradictory findings with previous literature.

Though preliminary, these findings provide a strong framework for the development of future research planning. The effect sizes provided by the current study can be used to conduct power analyses for future, larger studies in this area. Such larger studies will allow for more powerful tests, leading to crucial corroboration, or lack thereof, of the current patterns. Importantly, the current study utilized both qualitative and quantitative data, allowing for the description of both common and less common experiences, which might be lost in a purely quantitative study, as well as better understanding of factors that might be driving quantitative relationships. These qualitative patterns are prime targets for future ongoing quantitative study.

Finally, this work can inform treatment and recovery planning, either in response to the ongoing COVID-19 pandemic or to future treatment interruptions (whether driven by large-scale natural disasters or due to individual factors such moving). Worsened mental health strengthened the influence of substance cravings on later substance use, which is supported by a large literature [67–70]. Thus, we recommend that comorbid mental health conditions be tracked and treated as a prime focus of SUD treatment. Although treatment, meetings, and schedules are all important factors in recovery [3], interruptions did not reduce the effect of cravings on later substance use, likely because many shifted to online virtual formats. The COVID-19 pandemic has led to many suggesting expansion and continuation of online meetings [46–49]. Whereas some of our participants noted positives of online treatment and meetings, others highlighted the impersonal nature of these approaches. Thus, we recommend working to make online strategies better matched to individual needs, noting that some individuals might thrive in these approaches, whereas others might not.

Self-care may be a particularly key skill for individuals in recovery during interruptions to treatment. Self-care is often taught in SUD and general behavioral health treatment [54, 71] and could be practiced at-home during the early stages of the pandemic and beyond. Whereas other coping strategies might be interrupted (e.g., not being able to go to the movies or restaurants, be with friends/family), self-care strategies, such as reflection/prayer, journaling, or positive self-talk, could be engaged in regardless of surrounding circumstances [22], making them flexible during this most restrictive time. Such strategies likely work through behavioral mechanisms, such that they replace time spent seeking substances [52], and/or through more cognitive or emotional routes, increasing mood and decreasing stress and subsequent use [72]. Thus, the flexibility of these strategies, along with the multiple modes of action, make them particularly important strategies for recovery during this time and beyond.

### 4.2 Limitations

Limitations of this study include the small sample size and the limited racial and gender diversity in the sample. The small sample size limited the ability to conduct more complex, better controlled, and sophisticated statistical analyses, which should be done in future work. The risk for type I error also remains, as small sample size did not allow for correction without increased risk for type II error. Although the prospective nature of the study is a strength, limited power makes the conclusions here preliminary. Additionally, there are limitations to the measurements used. Previous 90-day substance use was self-reported by participants and therefore are limited by the accuracy of one’s memory, recall, and willingness to report. As data collection began during the onset of the pandemic, no validated interview instrument related to the pandemic existed; therefore, reliability and validity of our COVID-19 measure is in initial stages of development.

## 5.0 Conclusions

This mixed methods study examined initial reactions to the U.S. COVID-19 pandemic among individuals in recovery during the six weeks that corresponded with the initiation of state-mandated closings and how these reactions predicted later substance use outcomes. Such data would not be easy to gather again outside of another novel pandemic or similar wide-spread interruption to recovery services. The Substance Abuse and Mental Health Services Administration recently emphasized the need for prevention and treatment of SUD amid disaster preparedness and response and called for research to empirically identify important facilitators of recovery in such disasters [73]. The current study can inform how to best manage SUD recovery in response to future large-scale disruptions in recovery supports, such as when recovery supports might be restricted due to natural disasters (e.g., hurricanes, earthquakes) or individual life interruptions (e.g., when a person in recovery moves to a new city and must identify new recovery supports). Overall, findings suggest that though our participants in recovery experienced stressors characterized by the most disruptive and stringent COVID-19-related restrictions, they also demonstrated effective coping. These preliminary findings contribute to an emerging body of prospective work concerning the effects of the COVID-19 pandemic on SUD recovery and highlight facilitators of, in addition to barriers to, recovery during this time. Should substance use treatments be interrupted in the future, prioritizing treating mental health and encouraging self-care would be important points of planning and prevention. It is our hope that this study catalyzes future studies to better characterize ongoing patterns in larger, more properly powered, and diverse samples.

## Supporting information

S1 Fig Timeline of COVID-19 events in the state in which data were collected. (PDF)

S1 Table COVID-19 interview instrument. (PDF)

S1 Dataset Deidentified dataset. (SAV)
