# Supplementary figures and images for: Mixed methods prospective findings of the initial effects of the U.S. COVID-19 pandemic on individuals in recovery from substance use disorder
Source: PLoS One. 2022 Jul 1;17(7):e0270582. doi: 10.1371/journal.pone.0270582 (PMC9249176; doi:10.1371/journal.pone.0270582)

S1 Figure. Timeline of COVID-19 Events in the State in Which Data Were Collected.

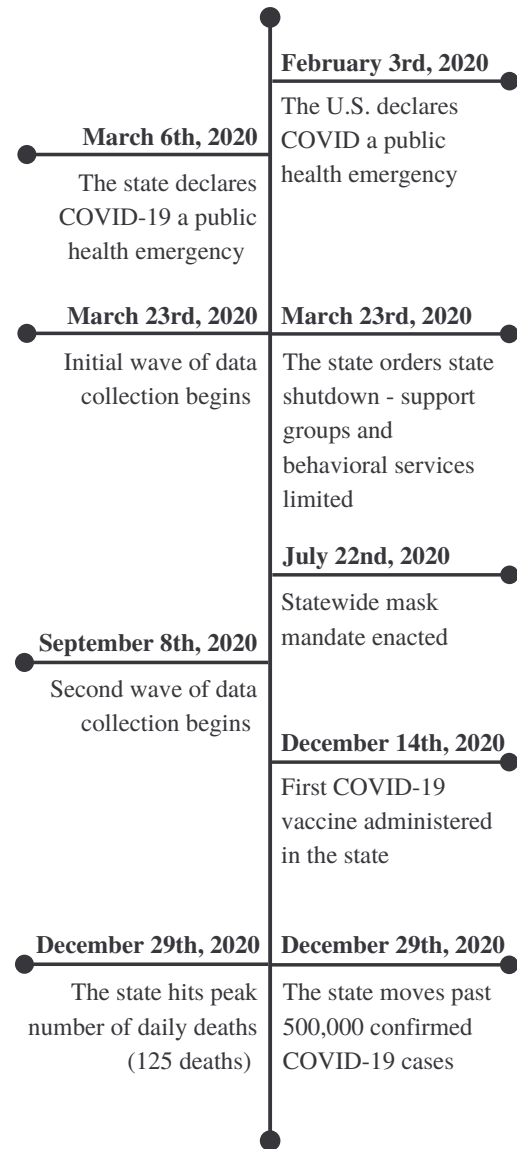

Supplement: S1 Fig — (PDF) [file pone.0270582.s001.pdf]
