# Supplementary material for: Mixed methods prospective findings of the initial effects of the U.S. COVID-19 pandemic on individuals in recovery from substance use disorder
Source: PLoS One. 2022 Jul 1;17(7):e0270582. doi: 10.1371/journal.pone.0270582 (PMC9249176; doi:10.1371/journal.pone.0270582)
Supplement: S1 Table — (PDF) [file pone.0270582.s002.pdf]

S2 Table. COVID-19 Interview Instrument.

Question	Response Type
How has the COVID-19 pandemic affected your everyday life?	Open ended
How worried are you about the COVID-19 pandemic?	0 (not worried) – 100 (extremely worried)
What do you think are the chances you will also become ill?	0 (% chance) – 100 (% chance)
Are you/have you been sick with COVID-19?	Yes / No
Do you or does someone close to you have a pre-existing condition that makes you or them at high risk for COVID-19?	Yes / No
Are you currently quarantined or isolated?	Yes / No
If yes, are you alone or with others?	Alone / With others
How has the COVID-19 pandemic affected your recovery or treatment plan?	Open ended
Have you noticed changes in substance use cravings?	Fewer cravings / No change / More cravings
If you are actively using, have you noticed changes in substance use?	Less use / No change / More use
Rate your current level of stress.	0 (not stressed) – 100 (extremely stressed)
Has your employment status changed?	Yes / No
If so, how?	Open ended
